# Oncological treatments have limited effects on the fertility prognosis in testicular cancer: A systematic review and meta‐analysis

**DOI:** 10.1111/andr.13741

**Published:** 2024-08-27

**Authors:** Janna Pape, Jancy Fernando, Dimitrios Megaritis, Susanna Weidlinger, Angela Vidal, Frédéric D. Birkhäuser, Tanya Karrer, Michael von Wolff

**Affiliations:** ^1^ Division of Gynecological Endocrinology and Reproductive Medicine University Women's Hospital Inselspital Bern University of Bern Bern Switzerland; ^2^ Frauenarztpraxis Langenthal Langenthal Switzerland; ^3^ Department of Urology Urology St. Anna Lucerne Switzerland; ^4^ Medical Library University Library Bern University of Bern Bern Switzerland

**Keywords:** azoospermia, cryosperm, fertility preservation, infertility, oncological treatment, testicular cancer

## Abstract

**Background:**

Testicular cancer is the most common solid tumour among young men in the reproductive phase. After completing cancer treatment, up to 77% of cancer survivors report an interest in paternity after completing cancer treatment. To preserve fertility, most guidelines recommend that physicians should counsel their patients about sperm cryopreservation before initiating gonadotoxic therapy. However, few studies have assessed fertility parameters after testicular cancer therapies over the last 20 years.

**Objectives:**

To close the gap of data regarding gonadotoxicity of testicular cancer therapies to enable more accurate counselling regarding fertility preservation.

**Materials and methods:**

A systematic literature search was conducted in Medline, Embase and Cochrane until December 2022. The systematic review included studies of men who had undergone all types of unilateral testicular cancer treatment, whereas the meta‐analysis excluded studies with unspecified treatments, less than 10 patients for outcome evaluation or rare tumours. Infertility (i.e. azoospermia, failure to achieve paternity or the usage of cryosperm) was defined as outcome.

**Results:**

The qualitative analysis included 30 studies with a total of 13,718 men after unilateral testicular cancer. Treatment comprised active surveillance after unilateral orchidectomy (32.7%), radiotherapy (23.1%), standard‐ or low‐dose chemotherapy (33.7%) and high‐dose chemotherapy (1.4%). Post‐treatment spermiograms were analysed in 17 studies. The quantitative synthesis included 23 studies, revealing an overall pooled prevalence of infertility (95% CI) of 14% (9%–21%). Azoospermia occurred in 8% (6%–12%). For good‐prognosis patients who received standard therapy, the overall prevalence of infertility was only 4% (2%–10%).

**Conclusion:**

So far, this very first meta‐analysis of overall infertility prevalence provides the best approximation of fertility prognosis for men who have undergone testicular cancer therapy. Despite the low prevalence of infertility, it is still recommended to undergo sperm cryopreservation because of the uncertainty of the subsequent therapy and the lack of large longitudinal data on individual treatment effects.

## 1 INTRODUCTION

Testicular cancer is the most common solid tumour among young men aged 15–45 years.^1^ Two thirds of patients are diagnosed in localized Stage I with a cure rate of over 95%. Even in metastasized disease, the chances of cure have significantly improved over the last two decades.^2^ More than 95% of patients become long‐term survivors,^3^ and up to 77% of cancer survivors report an interest in paternity after completing cancer treatment.^4^

Impaired fertility in testicular cancer patients is caused by both pre‐existing impaired spermatogenesis^5^ and treatment‐related gonadal toxicity: Radical inguinal orchiectomy is the main treatment for testicular cancer patients and can affect semen parameters and hormonal functions, leading to infertility.^6^ Cytotoxic agents such as alkylating agents and cisplatin compromise spermatogenesis at least temporarily or even induce permanent azoospermia depending on the drug combination and dosage.^7^, ^8^ Radiotherapy of the testicles with doses above 4 Gy can cause permanent germ cell defects, and 16–20 Gy might lead to irreversible azoospermia.^6^

Infertility has profound implications for the psychological and emotional well‐being of cancer survivors. Fertility preservation is now recognized as a crucial consideration prior to cancer treatment. Existing guidelines recommend that physicians should counsel their patients about fertility preservation measures before initiating gonadotoxic therapy.^9^, ^10^, ^11^, ^12^

For individual fertility counselling, it is important to estimate the risk of infertility because of the gonadal toxicity of cancer therapy. However, only few studies have assessed fertility parameters in testicular cancer patients after different testicular cancer therapies in the last 20 years: Compared with surveillance (i.e. unilateral orchidectomy only), cisplatin‐based chemotherapy and abdominal radiotherapy in standard dosage have been reported to decrease fertility to 3%–20% and 13%–30%, respectively.^13^ Given the limited longitudinal data available, this review with meta‐analysis aims to provide an approximation of fertility prognosis for fertility counselling in men who have undergone testicular cancer therapy.

Currently, the cryopreservation of ejaculated semen is the standard option for fertility preservation as a simple and effective method of fertility preservation in men.^14^, ^15^ However, sperm cryopreservation is not universally available,^16^, ^17^ and in some cases, its costs may not be fully covered, depending on the country's legislation and the perceived risk of infertility.^18^ Additionally, reported usage rates of stored material are often less than 10%,^15^, ^19^ indicating that many cryopreservations may be unnecessary and result in avoidable healthcare costs.

We have initiated a series of systematic reviews to establish a literature platform on the gonadal toxicity of different cancer group–specific therapies.^20^ This series is part of the project FertiTOX (www.fertitox.com), which also involves a prospective international multicentre data collection on the gonadal toxicity of cancer therapies in females and males.^21^

## 2 MATERIALS AND METHODS

### 2.1 Protocol registration

The study protocol was registered at the International Prospective Register of Systematic Reviews, PROSPERO (Registry number CRD42023384057). The Preferred Reporting Items for Systematic Reviews and Meta‐Analysis (PRISMA)^22^ was used.

### 2.2 Search strategy

A systematic literature search was conducted in Embase via Ovid, Medline ALL via Ovid and the Cochrane Database of Systematic Reviews and the Cochrane Central Register of Controlled Trials via Wiley in December 2022. An initial search strategy was developed in Embase by a medical information specialist and tested against a list of core references. After refinement and consultation, comprehensive search strategies were set up for each information source based on database‐specific controlled vocabulary (thesaurus terms/subject headings) and text words. Synonyms, acronyms and similar terms were included in the text word search. The search was limited to publications since 2000. The search concepts included all types of testicular cancer, oncological therapies (unilateral orchidectomy and surveillance, chemotherapy, radiotherapy) and gonadotoxic effects reflected by influences on fertility parameters. The Medline and Embase searches excluded animal‐only studies using a double‐negative search strategy based on the ‘humans only’ filters by Ovid. The detailed final search strategies are presented as a Supporting Information. Reference lists and bibliographies were scanned for relevant studies. References were imported into EndNote, and duplicates were removed.

### 2.3 Inclusion and exclusion criteria

The studies were assessed for inclusion by three investigators (JF, DM and JP) using the Covidence software (www.covidence.org). Original papers containing information on tumour type, tumour therapy and fertility results with numerical data enabling prevalence calculation were considered eligible. Clinically relevant gonadal toxicity was defined as infertility, including both azoospermia at least 12 months after oncological treatment and failure to achieve pregnancy after 12 months of regular unprotected sexual intercourse and/or usage of cryosperm. Studies with men after bilateral orchidectomy were excluded.

### 2.4 Data extraction

Three investigators (JF, DM and JP) independently abstracted and reviewed the extracted data in detail. Key variables of interest were: Characteristics of the study populations (age of patients at diagnosis and outcome, length of follow‐up, ethnicity), histology of testicular tumour, oncological treatment (surveillance after unilateral orchidectomy, dosage of chemo‐ and radiotherapy, combined therapies) and fertility parameters (spermiograms before and after therapy, attempts to conceive, usage of cryosperm). Discrepancies were discussed and resolved by consensus.

### 2.5 Quality assessment

The quality of the studies was evaluated using the Newcastle–Ottawa scale.^23^ The scoring of each study was based on three parameters: subject selection (0–4 stars), comparability (0–2 stars) and study outcome (0–3 stars). The final rating was calculated as follows: The study quality was classified as good, fair or poor based on the number of stars in the selection, comparability and outcome/exposure domains. Good‐quality studies received three or four stars in the selection domain, one or two stars in the comparability domain and two or three stars in the outcome/exposure domain. Fair‐quality studies received two stars in the selection domain, one or two stars in the comparability domain and two or three stars in the outcome/exposure domain. Poor‐quality studies received zero or one star in the selection domain, zero stars in the comparability domain or zero or one stars in the outcome/exposure domain.

The risk of bias was independently assessed by JF, DM and JP, and any disagreements were resolved by consensus.

### 2.6 Data synthesis

The systematic review aimed to determine the prevalence of infertility in men who underwent oncological treatments for unilateral testicular cancer. Infertility prevalence was calculated by dividing the number of patients who met the criteria for infertility by the number of patients at risk for infertility in each study. The pooled prevalence was analysed using the ‘metafor’ function in R software (R Core Team 2013). To examine heterogeneity, we used Cohen's *Q* statistic and *I* ^2^ statistic. In the presence of high heterogeneity, we employed random effects models. To ensure clinically meaningful estimates in the meta‐analysis, we excluded studies with unspecified treatments, less than 10 patients for outcome evaluation, or rare tumours. We conducted a subgroup analysis in good‐prognosis patients based on the International Germ Cell Cancer Consensus Group (IGCCCG) risk classification.^24^

## 3 RESULTS

### 3.1 Systematic review results

**FIGURE 1:**
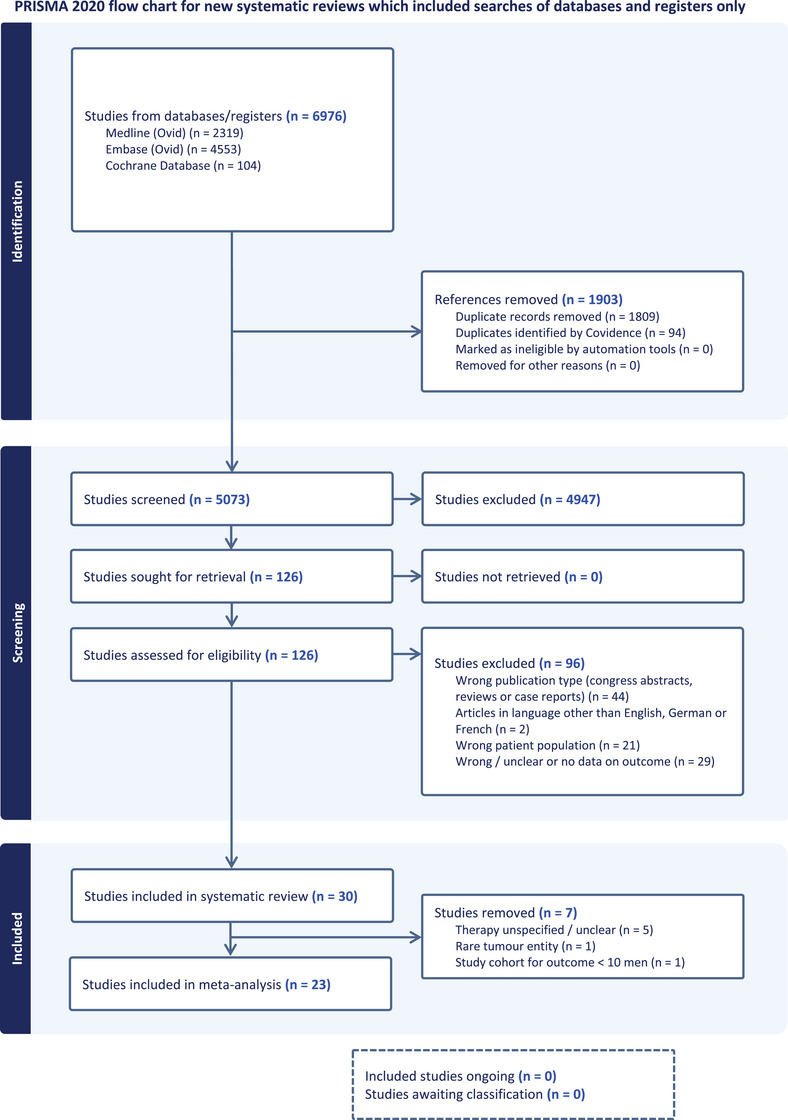
Preferred Reporting Items for Systematic Reviews and Meta‐Analysis (PRISMA) flow diagram. Flowchart of the literature search and selection process.

After screening the abstracts and full texts, 126 studies were considered. Of these, 96 studies were excluded for not meeting the criteria. Finally, only 30 articles met our inclusion criteria and were included in the systematic review (Figure 1).

#### 3.1.1 Study characteristics

Characteristics of the study populations are summarized in Table 1. The studies included in this analysis were registry analyses, with or without patient surveys (*n* = 12), as well as prospective (*n* = 10) or retrospective observational studies (*n* = 8). Only one study^25^ compared fertility outcomes with an age‐matched control group of healthy men from the normal population. Except for the high‐quality study conducted by Bandak et al. in 2022,^25^ most of the studies were rated as poor (*n* = 23) or fair (*n* = 6) in terms of methodological quality. This was mainly because of the lack of a comparison group or selection bias in questionnaire‐based studies (Table 2).

**TABLE 1:** Characteristics of the included studies.

First author, year	Country	Study design	Number of patients (time of therapy)	Age at diagnosis/therapy	Age at outcome/evaluation	Follow‐up (years)	Type of tumour (%)	Surgery + surveillance (%)	CT (%)	RT (%)	Spermiogram (before therapy [%])	Spermiogram (after therapy [%])	Hormonal analysis	Infertility (%)	Comments
Huyghe et al., 2001^66^	France	Survey	314 (1978–1998) 293 eligible	NT	28 (15–52)	NT	Seminoma: 107 (36.5) Non‐seminoma: 186 (63.5)	NT	188 (64.2) High‐dose: 21 (7.2) Unknown: 12 (4.1)	101 (34)	Total: 293 AS: 18 (6.1) OS: 64 (21.8) NS: 211 (72.0)	No	No	Total: 47/138 (34.1)* 9/138 (6.5) with ART	*Calculated from men without achieving paternity or needing ART, subset of men who try to conceive
Schreiber et al., 2001^42^	Germany	Retrospective	51 (therapy since 1991)	27.3 (5.3)	NT	4 (2–6)	Seminoma: 11 (21.6) Non‐seminoma: 40 (78.4)	27 (52.9) ±RPLND 12 (23.5)	10 (19.6) ±/−RPLND	2 (3.9)	Total: 51 AS: 0 (0)	Total: 30 AS: 0 (0) NS: 30 (100)	FSH Inhibin B	0/30 (0)*	*Calculated from men with AS after follow‐up (mean 4 years)
Daudin et al., 2002^43^	France	Retrospective	44	26.7 (5.8)	NT	2.1 (0.3–9.4)	Unspecified	0 (0)	44 (100) 2 cycles: 21 (47.7) 3 cycles: 16 (36.4) 4 cycles: 7 (15.9)	0 (0)	Total: 44 AS: 0 (0) OS: 24 (54.4) NS: 20 (46)	Total: 44 AS: 0 (0) OS: 28 (63.6) NS: 16 (36)	No	0/44 (0)*	*Calculated from men with AS at follow‐up
Huyghe et al., 2002^52^	France	Survey	489 (1978–1998) 446 eligible	NT	30 (14–73)	6.9 (3–26.3)	Unspecified	18 (4.0)	112 (25.1) ±RPLND: 44 (9.9) ±High‐dose or ±/−RT: 87 (19.5)	158 (35.4)	No	No	No	65/170 (38.2)*	*Calculated from men without achieving paternity, subset of men who try to conceive
Jacobsen et al., 2002^44^	Norway	Retrospective	174 (1980–1999)	28 (17–56)	NT	8.3 (3.8–18.1)	Seminoma: 13 (7.5) Non‐seminoma: 161 (92.5)	83 (47.7)	90 (51.7) (All ±RPLND or RT)	1 (0.6)	No	Total: 147 AS: 1 (0.7)	FSH LH Testosterone	20/77 (26.0)*	*Calculated from men without achieving paternity, subset of men who try to conceive
Tomomasa et al., 2002^31^	Japan	Prospective	18 (1986–2001)	29.5 (19–45)	NT	2.5 (1–4)	Seminoma: 10 (55.6) Non‐seminoma: 8 (44.4)	10 (55.6)	8 (44.4) All high‐dose	0 (0)	Total: 6 AS: 1 (16.6) OS: 5 (83.3) NS: 0 (0)	Total: 6 AS: 1 (16.6) OS: 4 (66.6) NS: 1 (16.7)	FSH LH Testosterone Prolactin	0/5 (0)*	*Calculated from men with AS after end of follow‐up (7 years), delayed restoration (>3 years)
Spermon et al., 2003^50^	Netherlands	Survey	305 (1982–1999) 226 eligible	31.7 (17.4–70.0)	32.1 (22.1–58.7)	>1.5	Seminoma: 54 (23.9) Non‐seminoma: 172 (76.1)	20 (8.8) ±RPLND 44 (19.5)	44 (19.5) ±RPLND 82 (36.3)	36 (15.9)	Total: 75 AS: 10 (13.3) OS: 43 (57.3) NS: 22 (29.3)	No	No	42/88 (47.4)* 8/88 (9.1) with ART	*Calculated from men without achieving paternity or needing ART, subset from who try to conceive
Eberhard et al., 2004^34^	Sweden	Prospective	112	29 (<50)	NT	5	Seminoma: 40 (35.7) Non‐seminoma: 72 (64.3)	0 (0)	74 (66.1) 1–2 cycles: 32 (28.6) ≥2 cycles: 42 (37.5)	38 (33.9)	No	Total: 53 AS: 5 (9.4)	No	5/53 (9.4)*	*Calculated from men with AS at follow‐up (5 years)
Huyghe et al., 2004^26^	France	Survey	451 (1978–1999)	NT	30.5 (14–73)	8.4 (3–26.5)	Seminoma: 216 (47.9) Non‐seminoma: 253 (56.1)	21 (4.7) ±/−RPLND	143 (31.7) ±High‐dose CT or ±/−RT: 116 (25.7)	171 (37.9)	No	No	No	Total: 54/170 (31.7)* 7/170 (4.1) used cryosperm	*Calculated from men without achieving paternity or using cryosperm, subset of men who try to conceive
Pectasides et al., 2004^48^	Greece	Prospective	173 (1986–1996) 69 eligible	27 (17–39)	NT	2.9 (0.9–5.2)	Seminoma: 3 (4.3) Non‐seminoma: 66 (95.7)	0 (0)	69 (100) 2 cycles: 25 (36.2) 3–4 cycles: 39 (56.5) ≥4 cycles: 5 (5.8), incl. 8 (68.9) ± RPLND	0 (0)	Total: 69 AS: 8 (11.6)	Total: 69 AS: 8 (11.6) OS: 23 (33.3) NS: 38 (55.1)	FSH LH Testosterone	8/69 (11.6)*	*Calculated from men with AS after follow‐up (mean 2.9 years)
Brydoy et al., 2005^13^	Norway	Survey	1814 (1980–1994) 1433 eligible for paternity analysis	32 (15–64)	43 (23–75)	10.6 (3–20)	Seminoma: 718 (50.1) Non‐seminoma: 715 (49.9)	119 (8.3)	551 (38.4) ±RPLND: 365 (25.5) High‐dose: 104 (7.3)	610 (42.6)	No	No	No	Total: 191/564 (33.8)* 100/459 (21.8) with ART	*Calculated from men without achieving paternity, subset of men who try to conceive
Huddart et al., 2005^67^	The UK	Retrospective	1603 (1982–1992) 680 eligible	31.7 (10–82)	44 (23–78)	10.2 (0.01–20.3)	Seminoma: 292 (43) Non‐seminoma: 388 (47)	169 (24.9)	272 (40.0) ±RT: 81 (11.9)	158 (23.2)	No	No	FSH LH Testosterone	Total: 48/207 (23.2)* 10/207 (4.8) with ART	*Calculated from men without achieving paternity or needing ART, subset of men who try to conceive
Magelssen et al., 2005^27^	Norway	Retrospective	1388 (1983–2002)	30.3 (14.8–49.7)	NT	8.4 (0.01–20.8)	Seminoma: 629 (45) Non‐seminoma: 759 (55)	256 (53) ±RPLND: 45 (5)	635 (45.7) Standard: 549 (40) High‐dose: 86 (6)	450 (32)	Total: 1032 AS: 94 (9)	No	FSH	29/414 (7) used cryosperm	*Calculated from men using cryosperm, subset of men who try to conceive
Gandini et al., 2006^36^	Italy	Prospective	166	28.3 (4.6)	NT	2	Seminoma: 95 (57.2) Non‐seminoma: 71 (42.8)	0 (0)	71 (42.8)	95 (57.2)	Total: 166 AS: 0 (0)	Total: 90 AS: 4 (4.4)	No	4/90 (4.4)*	*Calculated from men with AS at follow‐up (2 years)
Spermon et al., 2006^37^	Netherlands	Prospective	22	31.2 (22.2–41.6)	48.2 (18.4–84.8)	4 (1.5–7.1)	Seminoma: 3 (13.6) Non‐seminoma: 19 (86.4)	0 (0)	22 (100)	0 (0)	Total: 22 AS: 0 (0) NS: 22 (100)	Total: 22 AS: 2 (9.1) OS: 7 (31.8) NS: 13 (59.1)	FSH LH Testosterone	2/22 (9.1)*	*Calculated from men with AS after follow‐up (mean 4 years)
Girasole et al., 2007^28^	The USA	Survey	330 (1994–2004) 129 responder	NT	34.1 (14–76)	NT	Unspecified	40 (31.0)	67 (51.9)	21 (16.3)	No	No	No	Total: 4/87 (4.6)* 3/31 (9.7) used cryosperm	*Calculated from men without achieving paternity or who have used cyrosperm, subset of men who try to conceive
Huyghe et al., 2007^45^	France	Survey	17	NT	32 (24–51)	7 (3–14.3)	Leydig cell: 17 (100)	17 (100)	?	?	Total: 17 NS: 5 (29.4)	No	No	3/10 (30.0)*	*Calculated from men without achieving paternity, subset of men who try to conceive
Pectasides et al., 2009^35^	Greece	Prospective	30 (1997–2003)	29 (17–62)	NT	5.3 (0.9–8.4)	Non‐seminoma: 30 (100)	0 (0)	30 (100)	0 (0)	Total: 21 AS: 3 (14.2) OS: 7 (33.3) NS: 11 (52.3)	Total: 21 AS: 4 (19) OS: 7 (33.3) NS: 10 (47)	FSH LH Testosterone	4/21 (19)*	*Calculated from men with AS after follow‐up (mean 5.3 years)
Brydoy et al., 2010^56^	Norway	Survey	1814 (1980–1994) 1462 respond 106 eligible	26 (15–37)	38 (25–53)	12 (5–20)	Seminoma: 4 (4) Non‐seminoma: 102 (96)	0 (0)	106 (100) (All ±RPLND)	0 (0)	No	Total: 71 AS: 14 (19.7) OS: 17 (23.9) NS: 40 (56.3)	FSH LH Testosterone	Total: 21/106 (19.8)* 6/106 (5.7) with ART	*Calculated from men needing ART (*n* = 6) and men without successful paternity (*n* = 15) after treatment, subset of men who try to conceive
Matos et al., 2010^51^	Slovenia	Survey	490 men (1976‐2002) 297 eligible	26 (16‐39)	26 (20–39)	10 (3‐39)	Seminoma: 79 (26.6) Non‐seminoma 218 (73.4)	5 (1.7) ±RPLND: 54 (18.2)	85 (28.6) ±RPLND: 116 (39)	37 (12.5)	No	No	No	76/150 (50.7)*	*Calculated from men without achieving paternity, subset of men who try to conceive
Brydoy et al., 2012^49^	Norway	Survey	1814 (1980–1994) 1191 eligible for analysis	31 (15–58)	43 (23–65)	11 (4–22)	Seminoma: 579 (48.6) Non‐seminoma: 613 (51.5)	232 (19)	474 (39.8) ≤4 cycles: 341 (28.6) ≥4 cycles: 85 (7.1) ±RT: 48 (4)	306 (25.7)	No	Total: 342 AS: 51 (14.9) OS: 98 (28.7) NS: 193 (56.4)	FSH Inhibin B	166/486 (34.2)*	*Calculated from men without paternity, subset of men who try to conceive (incl. 46 (9%) with dry ejaculation)
Bujan et al., 2013^38^	France	Retrospective	129	30.9 (22–44)	32.9 (24–46)	2	Seminoma: 70 (54.2) Non‐seminoma: 59 (45.7)	0 (0)	62 (48.1) 1–2 cycles: 17 (13.2) 3–4 cycles: 45 (34.9)	67 (51.9)	Total: 129 AS: 0 (0)	Total: 129 AS: 3 (2.3)	No	3/129 (2.3)*	*Calculated from men with AS 2 years after treatment
Di Bisceglie et al., 2013^39^	Italy	Prospective	261 (2000–2008)	27.9 (0.6)	30.9 (0.6)	3	Seminoma: 107 (41.0) Non‐seminoma: 154 (59.0)	0 (0)	154 (59.0)	107 (41.0)	Total: 261 AS: 0 (0)	Total: 261 AS: 0 (0)	FSH, Inhibin B	0/261 (0)*	*Calculated from men with AS at follow‐up (3 years)
Suzuki et al., 2013^33^	Japan	Prospective	49 (1991–2006)	30.6 (5.8)	NT	7	Unspecified	0 (0)	49 (100) Standard: 45 (91.8) High‐dose: 4 (8.2)	0 (0)	Total: 49 AS: 5 (10.2) OS: 38 (77.6) NS: 6 (12.2)	Total: 49 (100) AS: 3 (6.1)	No	3/49 (6.1)*	*Calculated from men with AS after end of follow‐up, delayed restoration (>3 years) in high‐dose CT
Isaksson et al., 2014^40^	Sweden	Prospective	459 men 217 included	32.6 (18–50)	NT	3–5	Seminoma: 100 (46.1) Non‐seminoma: 117 (2.2)	23 (10.6)	132 (60.8) 1–2 cycles: 74 (34.1) 3–4 cycles: 50 (23.0) High‐dose ±/−RT: 8 (3.7)	62 (28.6)	Total: 119 AS: 2 (1.7)	Total: 117 AS: 6 (5.1)	Inhibin B	9/117 (7.7)*	*Calculated from men with AS after follow‐up (3–5 years)
Molnár et al., 2014^29^	Hungary	Survey	86 59 eligible	27 (16–41)	32 (21–47)	4.6 (3.8)	Seminoma: 27 (45.8) Non‐seminoma: 32 (54.2)	0 (0)	33 (55.9) ±RT: 3 (5.1)	23 (39)	Total: 59 AS: 12 (20.3) OS: 12 (20.3) NS: 35 (59.3)	No	No	25/38 (65.8)* 7/38 (18.4) used cryosperm	*Calculated from men without achieving paternity or using cryosperm, subset of men who try to conceive
Ghezzi et al., 2016^41^	Italy	Retrospective	212	33.4 (2.6)	34.4 (2.6)	2	Unspecified	58 (27.4)	100 (47.2) 1× carboplatin: 54 (25.5)	0 (0)	Total: 212 AS: 0 (0)	Total: 212 AS: 0 (0)	FSH LH Testosterone	0/212 (0)*	*Calculated from men with AS at follow‐up (2 years)
Namekawa et al., 2016^32^	Japan	Prospective	35 (1982–2001)	26 (17–34)	NT	1.1 (5.6)	Seminoma: 9 (25.7) Non‐semonima: 26 (74.3)	0 (0)	35 (100) High‐dose: 9 (25.7) Low‐dose: 26 (74.3) ±RPLND: 15 (42.9)	0 (0)	Total: 35 AS: 0 (0)	Total: 35 AS: 5 (14.3) NS: 30 (85.7)	FSH LH Testosterone	5/35 (14.3)*	*Calculated from men with AS after follow‐up (3.5 years)
Yamashita et al., 2021^30^	Japan	Survey	567 (2018–2019)	36 (22–50)	43 (27–59)	5.2 (1–14.9)	Seminoma: 256 (45.1%) Non‐seminoma: 294 (51.9%) Unknown: 17 (3.0%)	169 (29.8) (All ±/−RPLND)	398 (70.2) (All ±/−RPLND	0 (0)	No	No	No	28/121 (23.1)*	*Calculated from men using cryosperm, subset from men who try to conceive
Bandak et al., 2022^25^	Denmark	Retrospective	4846 (1984–2007)/with access to ART: 2648 (1995–2007)	33.8 (27.7–41.0)	NT	30	Seminoma: 2598 (53.6) Non‐seminoma: 2248 (46.4)	3225 (66.5)	1036 (21.3) ±RPLND: 557 (11.5)	729 (15.0)	No	No	No	178/2648 (6.7)*	*Calculated from men needing ART after treatment, subset of men with access to ART because of male infertility

*Note*: Summary of the cohort studies investigating the prevalence of infertility after unilateral testicular cancer therapies. The studies are sorted by year of publication. Age and duration of follow‐up are given as years with a mean (SD) or range where such data are available.

Abbreviations: ART, assisted reproduction technology; AS, azoospermia; CT, chemotherapy; NR, not reported; NS, normozoospermia; OS, oligozoospermia; RPLND, retroperitoneal lymph node dissection; RT, radiotherapy.

**TABLE 2:** Bias screening.

	Selection				Comparability		Outcome					
Study	Representativeness of exposed cohort	Selection of non‐exposed cohort	Ascertainment of exposure	Outcome of interest not present at start of study	Comparability of cohorts in main factors	Comparability of cohorts in additional factors	Assessment of outcome	Sufficient length of follow‐up for outcomes to occur	Adequacy of follow‐up of cohorts	Total	Quality assessment	Comments
Huyghe et al., 2001^66^	★	–	**★**	–	**–**	–	**–**	**★**	**–**	3/9	Poor	Survey
Schreiber et al., 2001^42^	**★**	–	**★**	**★**	**–**	–	**★**	**★**	**–**	5/9	Poor	No comparison group
Daudin et al., 2002^43^	**★**	–	**★**	**–**	–	–	**★**	**★**	**★**	5/9	Poor	No comparison group
Huyghe et al., 2002^52^	**★**	–	**★**	**★**	**–**	–	**–**	**★**	**–**	4/9	Poor	Survey
Jacobsen et al., 2002^44^	**★**	–	**★**	**★**	**★**	–	**★**	**★**	**–**	6/9	Fair	
Tomomasa et al., 2002^31^	**★**	–	**★**	**★**	**–**	–	**★**	**★**	**–**	5/9	Poor	No comparison group
Spermon et al., 2003^50^	**★**	–	**★**	–	**–**	–	**★**	**★**	**–**	4/9	Poor	Survey
Eberhard et al., 2004^34^	**★**	–	**★**	–	–	–	**★**	**★**	–	4/9	Poor	No comparison group
Huyghe et al., 2004^26^	**★**	–	**★**	**★**	**–**	–	**–**	**★**	**–**	4/9	Poor	Survey
Pectasides et al., 2004^48^	**★**	–	**★**	**★**	**–**	–	**★**	**★**	**–**	5/9	Poor	No comparison group
Brydoy et al., 2005^13^	**★**	–	**★**	–	**–**	–	**–**	**★**	**–**	3/9	Poor	Survey
Huddart et al., 2005^67^	**★**	–	**★**	–	**★**	–	**–**	**★**	**★**	5/9	Fair	
Magelssen et al., 2005^27^	**★**	–	**★**	**★**	**★**	–	**–**	**★**	**–**	5/9	Poor	No data on attempts of post‐treatment paternity
Gandini et al., 2006^36^	**★**	–	**★**	**★**	**★**	–	**★**	**★**	–	6/9	Fair	
Spermon et al., 2006^37^	**★**	–	**★**	**★**	**–**	–	**★**	**★**	**–**	5/9	Poor	No comparison group
Girasole et al., 2007^28^	**★**	–	**★**	–	**–**	–	**–**	**★**	**–**	3/9	Poor	Survey
Huyghe et al., 2007^45^	**★**	–	**★**	**★**	**–**	–	**–**	**★**	**–**	4/9	Poor	Survey
Pectasides et al., 2009^35^	**★**	–	**★**	**★**	**–**	–	**★**	**★**	**–**	5/9	Poor	No comparison group
Brydoy et al., 2010^56^	**★**	–	**★**	–	–	–	**–**	**★**	–	3/9	Poor	Survey
Matos et al., 2010^51^	**★**	–	**★**	**★**	**–**	–	**★**	**★**	**–**	5/9	Poor	Survey
Brydoy et al., 2012^49^	**★**	–	**★**	–	–	–	**–**	**★**	–	3/9	Poor	Survey
Bujan et al., 2013^38^	**★**	**–**	**★**	**★**	**★**	–	**★**	**★**	**★**	7/9	Fair	
Di Bisceglie et al., 2013^39^	**★**	–	**★**	**★**	–	–	**★**	**★**	**★**	6/9	Poor	No comparison group
Suzuki et al., 2013^33^	**★**	–	**★**	**★**	**–**	–	**★**	**★**	**–**	5/9	Poor	No comparison group
Isaksson et al., 2014^40^	**★**	–	**★**	**★**	**★**	–	**★**	**★**	**★**	7/9	Fair	
Molnár et al., 2014^29^	**★**	–	**★**	**★**	**–**	–	**★**	**★**	**–**	5/9	Poor	Survey
Ghezzi et al., 2016^41^	**★**	–	**★**	**★**	**★**	–	**★**	**★**	**★**	7/9	Fair	
Namekawa et al., 2016^32^	**★**	–	**★**	**★**	**–**	–	**★**	**★**	**–**	5/9	Poor	No comparison group
Yamashita et al., 2021^30^	**★**	–	**★**	**★**	**–**	–	**–**	**★**	**–**	4/9	Poor	Survey
Bandak et al., 2022^25^	**★**	**★**	**★**	**★**	**★**	–	**★**	**★**	**★**	8/9	Good	

*Note*: Newcastle‐Ottawa Quality Assessment Form for Cohort Studies.

In total, 13,718 men reported a history of unilateral testicular cancer, of which 6608 (48.1%) were eligible for fertility analysis. The sample sizes of the studies ranged from 17 to 4846 patients. The study reports on fertility parameters, including azoospermia after oncological treatment (*n* = 14) and failure to achieve pregnancy after ≥12 months of regular unprotected sexual intercourse (*n* = 13). Five studies^26^, ^27^, ^28^, ^29^, ^30^ evaluated the use of cryosperm to achieve paternity. Spermiograms were performed before treatment in 17 studies with a total of 2660 patients, which represents 19.4% of the entire study population. However, the corresponding results were not reported in the majority of the studies (74.5%). Spermiograms were performed in 17 studies after treatment, with a total of 1698 patients. Of these, 632 (37.2%) showed normozoospermia, 184 (10.8%) showed oligozoospermia and 112 (6.6%) showed azoospermia. The spermiogram results were not reported in nearly half of the patients (773/1.698 = 45.5%).

The patients included in the 30 studies were mainly from Europe (*n* = 25 studies); four studies^30^, ^31^, ^32^, ^33^ evaluated Asian populations, and only one study^28^ was conducted in the USA. The histology of the testicular tumours consisted of seminomas (6.020/13.718 = 43.9%), non‐seminomas (6.803/13.718 = 49.6%) and sex cord or stromal tumours (17/13.718 = 0.01%). The tumour was unspecified in 897 (6.5%) of the cancer survivors. The patients were mostly young, with a mean age of 29.7 years (range 10–82) at cancer diagnosis and a mean age of 35.4 years (range 14–85) at outcome evaluation. The studies had a long follow‐up (mean 6.5 years) ranging from 1 to 30 years. The treatment options included active surveillance after unilateral orchiectomy (32.7%), standard‐ or low‐dose chemotherapy (33.7%), high‐dose chemotherapy (1.4%) and radiotherapy (23.1%). The therapy was not defined in 8.4% of the study population.

#### 3.1.2 Prevalence of infertility

The prevalence of infertility in men with a history of unilateral testicular cancer and oncological treatment varies widely, ranging from 0% to 58.1%. Studies that included spermiograms after therapy^31^, ^32^, ^33^, ^34^, ^35^, ^36^, ^37^, ^38^, ^39^, ^40^, ^41^, ^42^, ^43^ and recent studies published between 2016 and 2022^25^, ^30^, ^32^, ^41^ tend to report lower rates of infertility, with some studies reporting rates as low as 19%.

### 3.2 Meta‐analysis results

To ensure clinically meaningful estimates, we excluded seven studies: Five of these studies did not provide detailed information on the applied therapies.^28^, ^29^, ^30^, ^42^, ^44^ One study only included Leydig cell tumours, which are rare and usually benign entities.^45^ Another study evaluated the fertility outcome in less than 10 patients (Figure 1).^31^

#### 3.2.1 Pooled overall prevalence of infertility after all types of treatments

**FIGURE 2:**
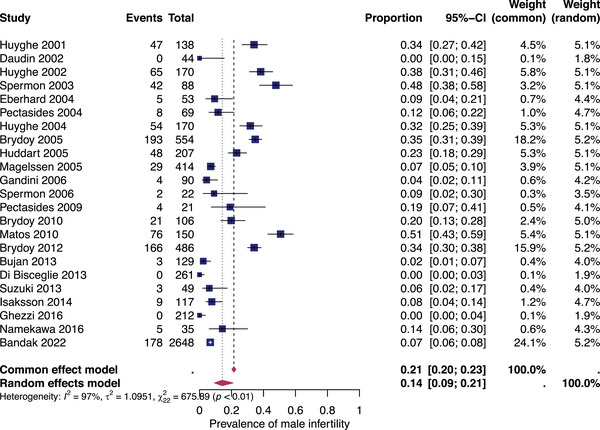
Pooled overall prevalence of suspected infertility. Forest plot of proportions and 95% confidence intervals (CIs) for studies evaluating the prevalence of infertility after all types of unilateral testicular cancer therapies. Blue squares for each study indicate the proportion, the size of the boxes indicates the weight of the study and the horizontal lines indicate the 95% CIs. The data in bold and pink diamond represent the pooled prevalence for post‐treatment infertility and 95% CIs. Overall estimates are shown in the fixed and random effects models.

Twenty‐three studies were included in the analysis of the overall prevalence of infertility. They involved patients who underwent various oncological treatments, including surveillance after orchidectomy with or without retroperitoneal lymph node dissection (RPLND), different types and dosages of platinum‐based chemotherapy and radiotherapy, and combinations of different therapies. Figure 2 shows the prevalence for each study and the summary prevalence. The prevalence of overall infertility was found to be 14% (95% CI: 9%–21%). Significant heterogeneity was observed among the studies (*I* ^2^ = 97, *p* < 0.01).

#### 3.2.2 Subgroup analysis: infertility in good‐prognosis patients

**FIGURE 3:**
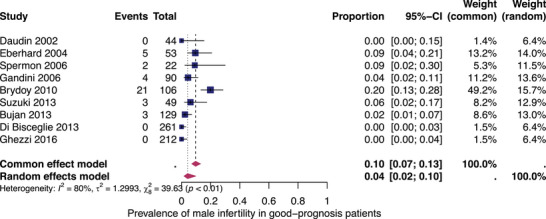
Pooled prevalence of infertility in good‐prognosis patients. Forest plot of proportions and 95% confidence intervals (CIs) for studies evaluating the prevalence of infertility in good‐prognosis patients after standard therapy (i.e. low‐ or standard‐dosed platin‐based chemotherapy up to four cycles and/or radiotherapy). Blue squares for each study indicate the proportion, the size of the boxes indicates the weight of the study and the horizontal lines indicate the 95% CI. The data in bold and pink diamond represent the pooled prevalence for post‐treatment infertility in good‐prognosis patients and 95% CIs. Overall estimates are shown in the fixed and random effects models.

Nine studies reported fertility outcomes in good‐prognosis patients based on the IGCCCG risk classification. The prevalence of infertility for each of these studies and the summary prevalence are shown in Figure 3. The overall prevalence of infertility in patients who received either low‐ or standard‐dose chemotherapy (with or without RPLND), low‐ or standard‐dose radiotherapy or combined radio‐chemotherapy was 4% (95% CI: 2%–10%). There was significant heterogeneity among the studies (*I* ^2^ = 80%, *p* < 0.01).

#### 3.2.3 Subgroup analysis: azoospermia after all types of treatments

**FIGURE 4:**
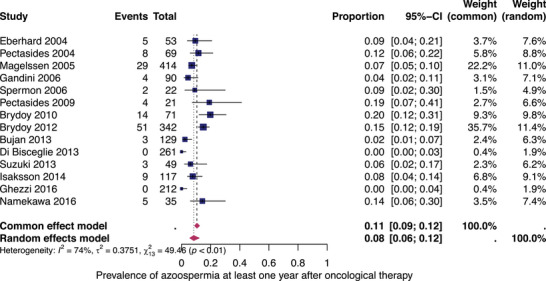
Pooled prevalence of azoospermia. Forest plot of proportions and 95% confidence intervals (CIs) for studies evaluating the prevalence of azoospermia after all types of unilateral testicular cancer therapies. Blue squares for each study indicate the proportion, the size of the boxes indicates the weight of the study and the horizontal lines indicate the 95% CIs. The data in bold and pink diamond represent the pooled prevalence for post‐treatment azoospermia and 95% CIs. Overall estimates are shown in the fixed and random effects models.

To eliminate the influence of female infertility factors, we analysed 14 eligible studies that reported the rates of azoospermia at least 12 months after oncological treatment. Figure 4 shows the prevalence for each of these studies and the summary prevalence. Azoospermia occurred in 8% (95% CI: 6%–12%) after all types of therapies. The studies exhibited significant heterogeneity (*I* ^2^ = 77%, *p* < 0.01).

**FIGURE 5:**
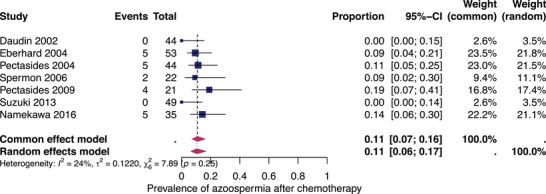
Pooled prevalence of azoospermia after orchiectomy and chemotherapy. Forest plot of proportions and 95% confidence intervals (CIs) for studies evaluating the prevalence of azoospermia after unilateral orchiectomy and all types of platinum‐based chemotherapy. Blue squares for each study indicate the proportion, the size of the boxes indicates the weight of the study and the horizontal lines indicate the 95% CIs. The data in bold and pink diamond represent the pooled prevalence for post‐treatment azoospermia and 95% CI. Overall estimates are shown in the fixed and random effects models.

Further stratification of the studies with patients who underwent primary unilateral orchiectomy followed by chemotherapy revealed a pooled prevalence of azoospermia of 11% (95% CI: 6%–17%) with low heterogeneity (*I* ^2^ = 24%) (Figure 5).

## 4 DISCUSSION

The aim of the systematic review and meta‐analysis was to analyse infertility outcomes in testicular cancer survivors to better counsel about fertility prognosis and the need to perform sperm cryopreservation. To our knowledge, this is the very first meta‐analysis to assess the pooled prevalence of infertility after oncological treatments in testicular cancer patients. A systematic review of 285 patients with testicular germ cell tumours analysed the oncological and functional outcomes after testis‑sparing surgery.^46^ However, this review included case reports with a high risk of bias and old studies before 2000. Additionally, the outcome criteria for infertility were not clearly defined.

As impaired semen parameters alone cannot predict fertility,^47^ we defined azoospermia, unsuccessful paternity and the use of cryosperm as infertility outcomes in our analysis.

Our study revealed the following important findings:

First, the overall pooled prevalence of infertility in the general population of testicular cancer survivors after at least 12 months following oncological treatment is generally low (14%, 95% CI: 8%–21%).

Second, the overall pooled prevalence of infertility in good‐prognosis patients who received standard therapy is even lower (4%, 95% CI: 2%–10%).

Third, the overall pooled prevalence of azoospermia as a clear male infertility outcome is only slightly higher, reaching 8% (95% CI: 6%–12%).

However, the quality of studies on this topic is poor, despite its high clinical relevance. There were only 10 prospective studies^31^, ^32^, ^33^, ^34^, ^35^, ^36^, ^37^, ^39^, ^40^, ^48^ and one retrospective study of good quality.^25^ Only 19% of the pooled study population had a spermiogram before therapy (either before orchiectomy or after orchiectomy but before the start of chemotherapy or radiotherapy), and an even smaller proportion (12%) had a spermiogram after completion of therapy. Subgroup analysis of individual treatments was not possible because of study cohorts of pre‐dominantly mixed therapies with aggregated outcomes.

Our findings on the good fertility prognosis in testicular cancer survivors are consistent with the results of the largest population‐based study to date, conducted by Bandak et al.^25^ In this study, the fertility of 4846 testicular cancer survivors treated between 1984 and 2007 was compared with that of an age‐matched healthy Danish population. Two thirds of the study population were in a surveillance programme after orchiectomy and had similar chances of achieving paternity. Following all types of testicular cancer therapies, the prevalence of infertility was 7% (95% CI: 6%–8%).

Our pooled prevalence of infertility is lower than the percentages reported in some of the included registry and questionnaire‐based studies.^13^, ^26^, ^29^, ^45^, ^49^, ^50^, ^51^, ^52^ This difference may be because of methodological weaknesses in the individual studies and the mixed types of therapies. The studies since 2013 have shown lower prevalence rates, which may be attributed to outdated data from earlier registry studies, selection bias in surveys and/or decreasing treatment intensity over the past 30 years.^53^ However, because of the non‐disaggregated data with mixed cohorts of good, intermediate and poor prognosis and different therapies, it was not possible to evaluate the individual treatment effects. The subgroup analysis of azoospermia following unilateral orchiectomy and chemotherapy (Figure 5) demonstrated a higher prevalence of azoospermia, with an estimated prevalence of 11% (95% CI: 6%–17%). However, it is important to note that different doses of chemotherapy were applied (i.e. one to more than four cycles of chemotherapy), whereas high‐dose chemotherapy may have greater gonadotoxic effects. However, only one study in clearly poor‐prognosis patients who received more than four cycles of platinum‐based chemotherapy^48^ showed an infertility prevalence of 19%.

During radiotherapy, the radiosensitive testicles are typically protected by gonadal shielding to minimize gonadotoxic effects caused by scatter radiation.^54^ Typically, scatter radiation doses are very low, reaching only 0.28% of the treatment dose.^55^ Our results in the good‐prognosis pooled cohort, which only included patients who underwent low‐ or standard‐dose chemotherapy with or without radiotherapy, are consistent with the minor effects on fertility.

In addition to chemotherapy and radiotherapy, RPLND may significantly affect fertility. According to a survey study by Brydoy et al.,^56^ only 11% of patients who underwent RPLND between 1980 and 1994 experienced antegrade ejaculation preservation after modified bilateral template RPLND, compared with 89% after the currently used nerve‐sparing operation technique.^57^ The high prevalence of infertility (nearly 20%) may have been caused by the old surgery technique used.

There is no data on pre‐pubertal testicular cancer survivors. However, pre‐pubertal tumours are very rare, usually benign and can be managed mainly with tumour excision. Therefore, we assume an excellent fertility prognosis.^58^

Although the evidence is limited, several guidelines^9^, ^10^, ^11^, ^12^, ^14^, ^59^, ^60^ suggest counselling male cancer patients about the possibility of infertility and conducting sperm cryopreservation for fertility preservation. Because of the limited and mostly inadequate studies, providing precise and age‐related data is not feasible. However, based on our meta‐analysis of clinically meaningful studies, it appears that fertility after treatment may be higher than expected. Therefore, only a small number of patients may require sperm cryopreservation.

Considering the positive fertility prognosis following testicular cancer therapy, it is worth questioning whether the general recommendation for sperm cryopreservation is still necessary, as it may result in unnecessary healthcare costs. Our meta‐analysis shows a low prevalence of azoospermia after all types of treatments. Additionally, Pacey et al.^61^ reported very low (<10%) utilization rates of banked spermatozoa, with the majority of samples being stored for long periods without being used. The low utilization rates of cryopreserved spermatozoa, combined with the high costs of cryopreservation and storage, may lead to a more restrictive implementation of sperm cryopreservation.^62^, ^63^

However, sperm cryopreservation before oncological treatment is non‐invasive and remains the most cost‐effective strategy for fertility preservation, across a range of possible costs associated with surgical sperm retrieval and in vitro fertilization/intracytoplasmic sperm injection.^62^ Therefore, we recommend sperm cryopreservation because of the uncertainty surrounding post‐surgery therapy and the lack of comprehensive longitudinal data on individual treatment effects.

Our study is based on well‐defined infertility parameters, including azoospermia, failure to achieve pregnancy after ≥12 months of regular unprotected sexual intercourse and/or the use of cryosperm. In addition, this meta‐analysis has a larger study population than previous cohort studies, resulting in higher statistical precision for testicular cancer patients. It is relevant to clinical practice as we applied strict exclusion criteria, such as excluding cohorts with less than 10 patients for outcome evaluation or rare tumour entities, and focused on good‐prognosis study cohorts, which comprise the majority of testicular cancer patients.^24^, ^64^ Therefore, our findings are applicable to current testicular cancer patients.

Even though our study strictly followed the recommendations to provide high‐quality summary reports of evidence, some limitations are evident:

First, the majority of the included studies were based on either questionnaire or register data with inherent selection bias: Only men who stated that they had a reproductive desire were eligible for fertility assessment, which might underestimate the true prevalence of infertility. Furthermore, it is possible that more intense treatments may lead to a decreased desire for fatherhood.

Second, failure to achieve a pregnancy after >12 months of regular unprotected sexual intercourse and/or the usage of cryosperm might be either because of inherent infertility already present before oncological treatment or because of female infertility. To assess infertility only because of male factors, we examined the prevalence of azoospermia at least 1 year after oncological treatment. However, it is important to note that spermatogenesis recovery can take more than 1 year,^65^ which may have led to an overestimation of the rate of azoospermia.

Third, the subgroup analysis was performed on mixed study cohorts with different therapies and aggregated outcome data, making it difficult to precisely estimate treatment factors. The lack of stratification of patient outcomes according to characteristics (type and dosage of chemotherapy, radiotherapy, surgery with/without RPLND) precluded further subgroup analyses. It was therefore not possible to evaluate the prevalence of azoospermia depending on the time after therapy, as the patients received spermiograms at different timepoints within the study cohorts.

Therefore, large studies are needed to obtain recent, age‐related and high‐quality fertility data and to improve patient counselling. The FertiTOX project, which involves approximately 60 centres across 3 countries, will serve as a model for such a study. Over a 4‐year period, data will be collected from 5000 females and 5000 males (www.fertitox.com).^21^ The project aims to conduct a retrospective systematic data analysis and a prospective cohort study to implement an internet platform on the gonadotoxicity of cancer therapies. This will improve the counselling of patients regarding fertility and fertility preservation by the network FertiPROTEKT. The data will be available to physicians globally from 2026 onwards.

In conclusion, this meta‐analysis provides the best available approximation for fertility prognosis after currently used testicular cancer therapies. Despite the relatively low prevalence of infertility, it is still recommended to undergo sperm cryopreservation because of the uncertainty surrounding post‐surgery therapy and the lack of comprehensive longitudinal data on individual treatment effects.

## AUTHOR CONTRIBUTIONS

**Janna Pape, Susanna Weidlinger, Angela Vidal** and **Michael von Wolff**: Conceptualization. **Dimitrios Megaritis, Jancy Fernando** and **Janna Pape**: Data curation. **Janna Pape, Dimitrios Megaritis** and **Jancy Fernando**: Formal analysis and investigation. **Janna Pape, Dimitrios Megaritis, Jancy Fernando** and **Tanya Karrer**: Methodology. **Michael von Wolff** and **Frédéric D. Birkhäuser**: Writing review and editing. **Michael von Wolff**: Funding acquisition and supervision. All authors reviewed the results and approved the final version of the manuscript.

## CONFLICT OF INTEREST STATEMENT

The authors declare no conflicts of interest.

## Supporting information


**Supplement S1** *Database Search Strategies*
Systematic literature search in Medline, Embase and Cochrane.

## Data Availability

The data that support the findings of this study are available from the corresponding author upon reasonable request.
