# Supplementary material for: Oncological treatments have limited effects on the fertility prognosis in testicular cancer: A systematic review and meta‐analysis
Source: Andrology. 2024 Aug 27;13(4):731–46. doi: 10.1111/andr.13741 (PMC12006886; doi:10.1111/andr.13741)
Supplement: Supplementary file 1 — Supplement S1 Database Search Strategies Systematic literature search in Medline, Embase and Cochrane. [file ANDR-13-731-s001.docx]

Database Search Strategies for Appendix

**Search date: December 14, 2022**

********************************************

**Ovid MEDLINE(R) ALL <1946 to December 13, 2022>**

Search Date: 14/12/2022

1 (((testes or testic* or "yolk sac*" or "tunica vaginalis" or (male*2 adj4 (germ cell* or germinal cell* or germinal or chorio*3))) adj3 (tumo?r* or cancer* or carcino* or neoplas* or chorio?carcinoma* or teratocarcinoma*)) or (male*2 adj4 ("malign* germinoma*" or teratoma*)) or "leydig cell tumo?r*" or androblastoma* or testiculoma* or "testis cancer*" or "testis tumo?r*").ti,ab. 21304

2 exp Testicular Neoplasms/dt, rt or "Neoplasms, Germ Cell and Embryonal"/dt, rt or Germinoma/dt, rt or Teratocarcinoma/dt, rt or Teratoma/dt, rt 7416

3 or/1-2 25677

4 (chemotherap* or chemo-therap* or chemoradiotherap* or chemo-radiotherap* or adjuvant drug therap* or carcinochemotherap* carcino-chemotherap* or antineoplastic agent* or anti-neoplastic agent* or antineoplastic drug* or anti-neoplastic drug* or antitumo?r agent* or anti-tumo?r agent* or antitumo?r drug* or anti-tumo?r drug* or anticancer* agent* or anti-cancer* agent* or anticancer* drug* or anti-cancer drug* or anticarcinogen* or anti-carcinogen* or anticancerogen* or anti-cancerogen* or ((cancer* or tumo?r* or neoplas*) adj3 treat*)).ti,ab,kf. 831375

5 exp Antineoplastic Agents/ or exp Combined Modality Therapy/ or exp Antineoplastic Combined Chemotherapy Protocols/ or exp chemotherapy, adjuvant/ or exp Antineoplastic Protocols/ or exp chemoradiotherapy/ or radioimmunotherapy/ or exp radiotherapy, adjuvant/ 1463737

6 (Radio-therap* or radiotherap* or radiationtherap* or chemoradiotherap* or radiochemotherap* or protontherap* or radiosurg* or radio-surg* or irradiation* or x-ray-therap* or therap* radiolog* or IMRT* or IORT* or radioimmunotherap* or radio-immuno-therap* or ((radiat* or irradiat* or radioisotope* or radio-isotope* or chemoradio or chemo-radio or radiochemo or radio-chemo or proton or x-ray or xray) adj2 (therap* or oncolog* or brachytherap* or brachy-therap*))).ti,ab,kf. 502694

7 exp Radiotherapy/ 204482

8 or/4-7 2238216

9 (fertili#ation* or fertility or fertile or fecund* or subfecund* or sub-fecund* or infecund* or infertility or sterility or subfert* or sub-fert* or anovularit* or gonad* or reproductive organ* or reproduction* or gamete-producing gland* or ovarian reserve* or ovary or ovaries or ovarian follicle* or oogenesis or oocyte* or amenorrhea* or premature menopaus* or early menopaus* or climacterium pr?ecox or Gonadotropin* or AMH or Anti-Mu?llerian Hormone* or Antimu?llerian Hormone* or Anti-Mu?llerian Factor* or Mu?llerian Inhibiting Hormone* or mu?llerian inhibitor* or FSH or Follicle Stimulating Hormone* or Folliculostimulating Hormone* or Follitropin or FSH-releasing hormone* or LH-FSH or testis or testes or testicle* or spermatogenes* or sperm* or semen or gametogenes* or hypogonadism* or hypo-gonadism* or "reproductive system*" or azoospermia* or spermatozoon*).ti,ab,kf. 757129

10 exp Fertility/ or exp Infertility/ or exp Gonads/ or Amenorrhea/ or anovulation/ or menopause, premature/ or Reproduction/ or Gametogenesis/ or Spermatozoa/ 371823

11 or/9-10 848753

12 3 and 8 and 11 4471

13 (exp animals/ or exp animal experimentation/ or exp models, animal/ or exp plants/ or exp fungi/) not humans/ 5496637

14 12 not 13 4349

15 limit 14 to yr="2000-current" 2319

********************************************

**Embase <1974 to 2022 December 14>**

Search Date: 14/12/2022

1 (((testes or testic* or "yolk sac*" or "tunica vaginalis" or (male*2 adj4 (germ cell* or germinal cell* or germinal or chorio*3))) adj3 (tumo?r* or cancer* or carcino* or neoplas* or chorio?carcinoma* or teratocarcinoma*)) or (male*2 adj4 ("malign* germinoma*" or teratoma*)) or "leydig cell tumo?r*" or androblastoma* or testiculoma* or "testis cancer*" or "testis tumo?r*").ti,ab. 26766

2 exp testis cancer/dt, rt [Drug Therapy, Radiotherapy] 5273

3 exp germ cell cancer/dr, rt [Drug Resistance, Radiotherapy] 2144

4 or/1-3 30356

5 (chemotherap* or chemo-therap* or chemoradiotherap* or chemo-radiotherap* or adjuvant drug therap* or carcinochemotherap* carcino-chemotherap* or antineoplastic agent* or anti-neoplastic agent* or antineoplastic drug* or anti-neoplastic drug* or antitumo?r agent* or anti-tumo?r agent* or antitumo?r drug* or anti-tumo?r drug* or anticancer* agent* or anti-cancer* agent* or anticancer* drug* or anti-cancer drug* or anticarcinogen* or anti-carcinogen* or anticancerogen* or anti-cancerogen* or ((cancer* or tumo?r* or neoplas*) adj3 treat*)).ti,ab,kf. 1248020

6 exp antineoplastic agent/ or exp multimodality cancer therapy/ or exp cancer chemotherapy/ or exp antineoplastic protocol/ 2822167

7 (Radio-therap* or radiotherap* or radiationtherap* or chemoradiotherap* or radiochemotherap* or protontherap* or radiosurg* or radio-surg* or irradiation* or x-ray-therap* or therap* radiolog* or IMRT* or IORT* or radioimmunotherap* or radio-immuno-therap* or ((radiat* or irradiat* or radioisotope* or radio-isotope* or chemoradio or chemo-radio or radiochemo or radio-chemo or proton or x-ray or xray) adj2 (therap* or oncolog* or brachytherap* or brachy-therap*))).ti,ab,kf. 687535

8 exp cancer radiotherapy/ 312482

9 or/5-8 3676695

10 (fertili#ation* or fertility or fertile or fecund* or subfecund* or sub-fecund* or infecund* or infertility or sterility or subfert* or sub-fert* or anovularit* or gonad* or reproductive organ* or reproduction* or gamete-producing gland* or ovarian reserve* or ovary or ovaries or ovarian follicle* or oogenesis or oocyte* or amenorrhea* or premature menopaus* or early menopaus* or climacterium pr?ecox or Gonadotropin* or AMH or Anti-Mu?llerian Hormone* or Antimu?llerian Hormone* or Anti-Mu?llerian Factor* or Mu?llerian Inhibiting Hormone* or mu?llerian inhibitor* or FSH or Follicle Stimulating Hormone* or Folliculostimulating Hormone* or Follitropin or FSH-releasing hormone* or LH-FSH or testis or testes or testicle* or spermatogenes* or sperm* or semen or gametogenes* or hypogonadism* or hypo-gonadism* or "reproductive system*" or azoospermia* or spermatozoon*).ti,ab,kf. 851027

11 exp fertility/ or exp infertility/ or exp semen analysis/ or exp gonad/ or exp amenorrhea/ or exp early menopause/ or reproduction/ or gametogenesis/ or spermatozoon/ 558358

12 or/10-11 993111

13 4 and 9 and 12 6544

14 (exp animal/ or exp invertebrate/ or nonhuman/ or animal experiment/ or animal tissue/ or animal model/ or exp plant/ or exp fungus/) not (exp human/ or human tissue/) 7509642

15 13 not 14 6334

16 limit 15 to yr="2000-current" 4553

********************************************

**Cochrane Library <1996 to present>**Search Date: 14/12/2022

#1 (((testes or testic* or germinal or (yolk NEXT sac*) or (tunica NEXT vaginalis) or (male NEAR/4 (germ NEXT cell*) or (germinal NEXT cell*) chorio*)) NEAR/3 (tumor* or tumour* or cancer* or carcino* or neoplas* or chorio?carcinoma* or teratocarcinoma*)) or (male NEAR/4(malign* NEXT germinoma*) or teratoma*) or (leydig NEXT cell NEXT tumor*) or (leydig NEXT cell NEXT tumour*) or androblastoma* or testiculoma* or (testis NEXT tumo?r*) or (testis NEXT cancer*)):ti,ab 470

#2 MeSH descriptor: [Testicular Neoplasms] explode all trees and with qualifier(s): [drug therapy - DT, radiotherapy - RT] 133

#3 MeSH descriptor: [Neoplasms, Germ Cell and Embryonal] this term only and with qualifier(s): [drug therapy - DT, radiotherapy - RT] 69

#4 MeSH descriptor: [Germinoma] this term only and with qualifier(s): [drug therapy - DT, radiotherapy - RT] 33

#5 MeSH descriptor: [Teratocarcinoma] this term only and with qualifier(s): [drug therapy - DT, radiotherapy - RT] 0

#6 MeSH descriptor: [Teratoma] this term only and with qualifier(s): [drug therapy - DT, radiotherapy - RT] 10

#7 #1 OR #3 OR #4 OR #5 OR #6 536

#8 (chemotherap* or chemo-therap* or chemoradiotherap* or chemo-radiotherap* or (adjuvant NEXT drug NEXT therap*) or carcinochemotherap* carcino-chemotherap* or (antineoplastic NEXT agent*) or (anti-neoplastic NEXT agent*) or (antineoplastic NEXT drug*) or (anti-neoplastic NEXT drug*) or (antitumor NEXT agent*) or (antitumour NEXT agent*) or (anti-tumor NEXT agent*) or (anti-tumour NEXT agent*) or (antitumor NEXT drug*) or (antitumour NEXT drug*) or (anti-tumor NEXT drug*) or (anti-tumour NEXT drug*) or (anticancer* NEXT agent*) or (anti-cancer* NEXT agent*) or (anticancer* NEXT drug*) or (anti-cancer NEXT drug*) or anticarcinogen* or anti-carcinogen* or anticancerogen* or anti-cancerogen* or ((cancer* or tumor* or tumour*or neoplas*) NEAR/3 treat*)):ti,ab,kw 113149

#9 [mh "Antineoplastic Agents"] OR [mh "Combined Modality Therapy"] OR [mh "Antineoplastic Combined Chemotherapy Protocols"] OR [mh "chemotherapy, adjuvant"] OR [mh "Antineoplastic Protocols"] OR [mh "Chemoradiotherapy"] OR [mh ^Radioimmunotherapy] OR [mh "Radiotherapy, Adjuvant"] 42445

#10 (Radio-therap* or radiotherap* or radiationtherap* or chemoradiotherap* or radiochemotherap* or protontherap* or radiosurg* or radio-surg* or irradiation* or x-ray-therap* or (therap* NEXT radiolog*) or IMRT? or IORT? or radioimmunotherap* or radio-immuno-therap* or ((radiat* or irradiat* or radioisotope* or radio-isotope* or chemoradio or chemo-radio or radiochemo or radio-chemo or proton or x-ray or xray) NEAR/2 (therap* or oncolog* or brachytherap* or brachy-therap*))):ti,ab,kw 49014

#11 [mh Radiotherapy] 6706

#12 #8 OR #9 OR #10 OR #11 149075

#13 (fertilization* or fertilisation* or fertility or fertile or fecund* or subfecund* or sub-fecund* or infecund* or infertility or sterility or subfert* or sub-fert* or anovularit* or gonad* or (reproductive NEXT organ*) or reproduction* or (gamete-producing NEXT gland*) or (ovarian NEXT reserve*) or ovary or ovaries or (ovarian NEXT follicle*) or oogenesis or oocyte* or amenorrhea* or (premature NEXT menopaus*) or (early NEXT menopaus*) or (climacterium NEXT praecox) or (climacterium NEXT precox) or Gonadotropin* or AMH or (Anti-Mullerian NEXT Hormone*) or (Anti-Muellerian NEXT Hormone*) or (Antimullerian NEXT Hormone*) or (Antimuellerian NEXT Hormone*) or (Anti-Mullerian NEXT Factor*) oe (Anti-Muellerian NEXT Factor*) or (Mullerian NEXT Inhibiting NEXT Hormone*) or (Muellerian NEXT Inhibiting NEXT Hormone*) or (mullerian NEXT inhibitor*) or (muellerian NEXT inhibitor*) or FSH or (Follicle NEXT Stimulating NEXT Hormone*) or (Folliculostimulating NEXT Hormone*) or Follitropin or (FSH-releasing NEXT hormone*) or LH-FSH or testis or testes or testicle? or spermatogenes* or sperm* or semen or gametogenes* or hypogonadism* or hypo-gonadism* or (reproductive NEXT system*) or azoospermia* or spermatozoon*):ti,ab,kw 40808

#14 [mh Fertility] OR [mh Infertility] OR [mh Gonads] OR [mh ^Amenorrhea] OR [mh ^anovulation] OR [mh ^"menopause, premature"] OR [mh ^Reproduction] OR [mh ^Gametogenesis] OR [mh ^Spermatozoa] 5740

#15 #13 OR #14 40868

#16 #7 AND #12 AND #15 with Cochrane Library publication date Between Jan 2000 and Dec 2022 104
